# Editorial: The Natural Killer Cell Interactome in the Tumor Microenvironment: Basic Concepts and Clinical Application

**DOI:** 10.3389/fimmu.2020.00872

**Published:** 2020-05-19

**Authors:** Alberto Anel, Julián Pardo, Martín Villalba

**Affiliations:** ^1^Apoptosis, Immunity and Cancer Group, Department of Biochemistry and Molecular and Cell Biology, Aragón Health Research Institute (IIS Aragón), University of Zaragoza, Zaragoza, Spain; ^2^Immunotherapy, Inflammation and Cancer Group, Aragón Health Research Institute (IIS Aragón), Zaragoza, Spain; ^3^Aragón i + D Foundation (ARAID), Government of Aragon, Zaragoza, Spain; ^4^IRMB, CHU Montpellier, Montpellier, France; ^5^IRMB, Univ Montpellier, INSERM, CHU Montpellier, CNRS, Montpellier, France

**Keywords:** NK cell, adoptive cell immunotherapy, off-the-shelf NK cells, ovarian cancer, pediatric tumors, checkpoint inhibitors, NK cell metabolism

NK cell activity is impaired in cancer patients, supporting the use of adoptive NK cell therapy, which is becoming a credible immunotherapy for hematological malignancies. This is even more so the case after the presentation of the first clinical study using anti-CD19 NK CAR cells, which showed a good clinical activity in the absence of toxicity. The possibility of targeting solid tumors is being studied by numerous laboratories, but the tumor microenvironment supports immune suppression. Unveiling the molecular and cellular mechanisms explaining this immunosuppression is a major goal.

For this special issue, we pointed to several specific subjects, such as the metabolic interactions of NK cells with tumor targets that would regulate their function or novel molecular strategies for generating off-the-shelf NK cell cancer immunotherapies. A total of 10 manuscripts have been accepted for publication, of which five are original research and five are reviews or minireviews.

Regarding the original research articles, Alvarez et al. have described the indirect contribution of the PD-1/PD-L1 system to the regulation of NK cell exhaustion using an *in vivo* murine model. They showed that a PD-1 blockade increased CD8^+^ T cell activation rates, which competed for IL-2 and resources with NK cells, retarding their activation but also their subsequent exhaustion. Federici et al. developed an exhaustive work characterizing NK-cell derived extracellular vesicles (NKEVs), separating true exosomes from microvesicles. They demonstrated that NKEVs supported immune activation, modulating the expression of key stimulatory molecules in monocytes and in T cells. They also demonstrated that the amount of NKEVs is reduced in the plasma of melanoma patients compared with healthy donors. The Martin Villalba's group, in collaboration with clinicians and the group of Alexia et al., investigated the antitumor effect of the new immunoadjuvant Polyoxidonium (PO) in breast cancer patients. They demonstrate that PO increases activation markers in dendritic cells, favoring T-cell activation. In addition, PO increased the degranulation capacity of NK cells, showing positive clinical effects in a percentage of patients. Diaz et al. performed an interesting clinical study on a cohort of 60 young and pediatric patients with hematological malignancies. Patients were engrafted with haploidentical stem cells after T- and B-cell depletion. Improved outcomes correlated with a rapid expansion of mature CD56^dim^ NK cells early after transplantation, suggesting a positive graft vs. leukemia effect. Directly entering in the off-the-shelf NK cells for cancer immunotherapy, Fernández et al. described the manufacturing in GMP conditions of allogeneic NKG2D CAR T cells. To avoid undesirable graft vs. host reactions, CD45^−^ memory T cells were used, and good expansions of active CAR T cells were obtained.

Regarding reviews, Nersesian et al. published a comprehensive work describing the typical immunosuppressive tumor microenvironment (TME) of ovarian tumors and how adoptive NK cell treatment can help to revert this situation, alone or in combination with other immunotherapies. In fact, at least nine clinical trials using this approach in ovarian cancer patients are currently ongoing. Terrén et al. put the point on the molecular mechanisms that explain the inactivation of NK cell function by the TME through the modulation of NK cell metabolism. This review has attracted interest, being the most read paper of the collection at the moment, with more than 6,000 views. Villalba et al. describe strategies to improve NK cell anti-tumoral function, including combination with mAbs to induce ADCC, “arming” NK cells with antibodies, or the use of metabolic drugs that could increase tumor sensitivity to activated NK cells. Burger et al. described a promising field for NK cell-based therapy; CAR NK cells would be used for the treatment of glioblastoma, with the tumor antigen HER2 as the main target at the moment. Finally, and in relation with the first original article mentioned at the beginning of this editorial, Lanuza et al. reviewed the available experimental evidences regarding the role of immune checkpoints in NK cell function during physiological and pathological (cancer) conditions, arriving at the conclusion that the main checkpoint molecules targeted in T cells (CTLA-4, PD-1, LAG-3) have low impact in NK cell function during physiological conditions. This might be an additional advantage when using adoptive allogeneic NK cell transfer in the treatment of cancer.

As stated in the presentation of the special issue, we can therefore consider that this collection of reviews and original articles highlights significant advances made in the field of NK cell-based therapy and indicate potential new useful directions, always keeping in mind the benefit of patients and the improvement of their quality of life.

## Author Contributions

All authors listed have made a substantial, direct and intellectual contribution to the work, and approved it for publication. AA wrote the first draft; MV revised the reviews section and JP revised the original papers section, proposing a final integrated text.

## Conflict of Interest

The authors declare that the research was conducted in the absence of any commercial or financial relationships that could be construed as a potential conflict of interest.

